# Hypertension With High Homocysteine Is Associated With Default Network Gray Matter Loss

**DOI:** 10.3389/fneur.2021.740819

**Published:** 2021-09-28

**Authors:** Yanliang Kong, Xin Li, Lina Chang, Yuwei Liu, Lin Jia, Lei Gao, Lijuan Ren

**Affiliations:** ^1^Department of Radiology, People's Hospital of Tongchuan City, Tongchuan, China; ^2^Department of Ultrasound, People's Hospital of Tongchuan City, Tongchuan, China; ^3^Department of Radiology, Zhongnan Hospital of Wuhan University, Wuhan, China

**Keywords:** hypertension, homocysteine, VBM, default mode network, cognitive impairment

## Abstract

Hypertension with high homocysteine (Hcy, ≥10 μmol/L) is also known as H-type hypertension (HHT) and proposed as an independent risk factor for stroke and cognitive impairment. Although previous studies have established the relationships among hypertension, Hcy levels, and cognitive impairment, how they affect brain neuroanatomy remains unclear. Thus, we aimed to investigate whether and to what extent hypertension and high Hcy may affect gray matter volume in 52 middle-aged HHT patients and 51 demographically matched normotensive subjects. Voxel-based morphological analysis suggested that HHT patients experienced significant gray matter loss in the default network. The default network atrophy was significantly correlated with Hcy level and global cognitive function. These findings provide, to our knowledge, novel insights into how HHT affects brain gray matter morphology through blood pressure and Hcy.

## Introduction

Hypertension is a well-known risk factor for stroke and cognitive impairment. In China, about 75% of primary hypertension is accompanied by high homocysteine (plasma Hcy ≥ 10 μmol/L) and is defined as H-type hypertension (HHT) (1). High Hcy is an independent risk factor for cerebrovascular events (2, 3) and cognitive impairment (4–7). Despite these prominent dual toxic effects, little is known about the neuroanatomical basis for cognitive impairment in HHT. Investigation upon the neuroanatomical basis may help to understand how dual cerebrovascular risk factors exacerbate the impact of brain morphological integrity, promoting a targeted prevention of risk factors and progression monitoring.

One potential neuroimaging biomarker that encodes cognitive impairment is integrity of the default mode network (DMN). The DMN was first proposed by Raichle et al. (8) and anatomically comprises the posterior cingulate/precuneus, bilateral inferior parietal lobule, lateral temporal, and hippocampus (9, 10). The DMN regions show less activity during goal-directed tasks and greater activity during resting state or several particular social cognitive tasks (9, 10). The DMN is considered to be a critical basis for advanced cognition of humans during development and evolution. Moreover, the DMN has been proposed as a neural biomarker for cognitive impairment in neuroimaging community. For example, DMN connectivity differentiates Alzheimer disease (11), mild cognitive impairment (12), mild traumatic injury (13, 14), and chemotherapy-related (15) cognitive impairment.

Previous studies on primary hypertension have shown evidence on the DMN. For example, primary hypertension is associated with increased coupling between the core and the dorsal medial subpart of the DMN during resting state (16) and abnormal spontaneous activity in DMN in a rodent model (17).

Etiologically, hypertension is a group of syndromes with high heterogeneity; each subgroup classification may represent different outcomes. Among the various types of hypertensions, HHT is the most common in China and has been suggested to predict more adverse consequences and outcomes (1). Essentially, HHT is a, at least, silent, cerebrovascular disease, often associated with higher silent infarcts, white matter hyperintensity (WMH), and gray matter loss (1, 18–24). These silent lesions represent physical dysconnectivity, compromised coordination between interconnected gray matter regions, and progressive atrophy itself. We know that cortical gray matter integrity is the neuroanatomical basis of large-scale brain networks that support human behaviors and cognition, and mounting evidence links gray matter atrophy to cognitive impairment (25–30). However, it is unclear that whether and to what extent HHT may affect the DMN gray matter morphometry.

Voxel-based morphometry (VBM) serves as a non-invasive, automatic framework for predicting brain disease and aging related model (31–34). Previously, it has been reported that hypertensive individuals experienced shrinkage of gray matter (35). Moreover, in dementia and mild cognitive impairment, the DMN appears to be the most impacted and has the ability to be a potential imaging biomarker (16, 36, 37). In both human and rodent studies, higher serum Hcy is associated with significant brain atrophy (38, 39); for more details, please see de Jager (40). Together, these evidences suggest that HHT is an adverse factor in the atrophy of DMN and cognitive performance and may interact with particular ways.

Here, we aimed to investigate whether HHT is associated with significant gray matter loss in the DMN and the relationships among the DMN atrophy, Hcy level, and global cognition. We hypothesized that HHT was associated with significant gray matter loss in the DMN, and Hcy level significantly mediated the relationship between DMN atrophy and global cognitive behavioral variables in patients with HHT.

## Materials and Methods

### Subjects

Fifty-two H-type hypertensive subjects (26 females and 26 males, mean age 53.20 ± 10.98 years) and 51 normotensive healthy adults (33 females and 18 males, mean age 50.84 ± 9.92 years) were enrolled in this study. Informed consent was obtained from all the participants, and the Ethical Committee for Human Research at the People's Hospital of Tongchuan City approved all the procedures in accordance with the *Declaration of Helsinki*.

All of subjects were screened and diagnosed by a physician. All the subjects received comprehensive neurological examination and blood and neurocognitive tests. The inclusion criteria for the HHT subjects were as follows: (a) 18–65 years; (b) right-handed; (c) conforming to diagnostic criteria for HHT, i.e., serum Hcy ≥ 10 μmol/L and clinical systolic pressure >140 mmHg and/or diastolic pressure >90 mmHg; (d) currently not receiving systemically antihypertensive treatment; and (e) no contraindications for magnetic resonance imaging (MRI) examination.

Exclusion criteria were as follows: (a) any previous cardiovascular or cerebrovascular event; (b) large-scale old cerebral infarction and cerebral hemorrhage; (c) major diseases, including psychiatric diseases, renal failure (serum creatinine level >176 mmol/L), liver damage (aspartate aminotransferase or alanine aminotransferase >40 IU/L), coronary heart disease, congestive heart failure, or history of malignant tumors; (d) structural abnormalities that could impair cognitive function; and (e) low education years (<6 years).

### Data Collection

We used a standardized questionnaire to record demographic information, life style, and medical history for each participant. Medical history including cardiovascular factors (hypertension, hyperlipidemia, stroke, coronary artery disease), diabetes complications, medication use, and age of diabetes onset were recorded using a standardized questionnaire. Body mass index (BMI) was calculated as weight/height (kg/m^2^). Neurocognitive tests were administered to examine global cognition function with the Mini-Mental State Examination (MMSE) (41, 42). Neurocognitive tests and questionnaire were administered by a trained neuropsychologist and nurse staff. The diastolic and systolic blood pressures were measured by a trained staff after resting for at least 5 min in a seated position.

### MRI Acquisition and Data Processing

Structural brain images, including 3D T1-weighted fast field echo (3D T1-FFE, repetition time = 8.29 ms, echo time = 3.81 ms, flip angle = 8°, slice thickness = 1 mm, 160 axial slices covering whole brain) high-resolution anatomical and T2-fluid-attenuated inversion recovery (FLAIR, repetition time = 8,000 ms, echo time = 140 ms, inversion time = 2,200 ms, flip angle = 90°, slice thickness = 1 mm, 160 axial slices covering whole brain) images, were acquired on a 1.5T MRI (Phillips, Achieva, Andover, MA, USA) scanner equipped with an eight-channel head coil.

T1 structural images were processed using the computational anatomy toolbox (CAT, version 12.6; http://www.neuro.uni-jena.de/cat/) implanted in SPM12 (www.fil.ion.ucl.ac.uk/spm). Briefly, T1 images were first visually inspected and manually reoriented to ensure image quality and whole brain covering and further segmented into gray matter, white matter, and cerebral spinal fluid, with the diffeomorphic anatomical registration through an exponentiated lie algebra (DARTEL) algorithm (31). The individual gray matter images were then spatially normalized into the Montreal Neurological Institute (MNI) standard space and modulated. The modulated and spatial normalized gray matter images were then smoothed with an isotropic Gaussian kernel of 8 mm full-width at half-maximum. The FLAIR images were used for manually calculating white matter hyperintensity (WMH) volume for each participant.

Two HHT subjects and one control were excluded from the analysis due to poor image quality or segment failure.

### Statistical Analysis

Demographic and clinical variables, Hcy, and cognitive tests were analyzed using SPSS 16 (SPSS Inc., USA). Categorical variables (including gender, diabetes, obesity, smoking, drinking, etc.) were tested using the chi-square test, while the remaining variables were tested using independent two-sample *t*-tests. The significance was set at *p* < 0.05. Differences/associations with a *p* < 0.05 were considered to be statistically significant.

### Mediation Analysis

Since prior research has suggested that Hcy level (X, independent variable) can predict cognitive functions (Y, dependent variable) (43), we considered to take the DMN gray matter volume as a mediator (M) and build a mediation model. The mediation analysis was conducted by using the Hayes Process macro (44) (http://www.afhayes.com/), which is an observed least-squares regressions path analysis modeling tool. We used a model 4, with the independent factor as Hcy level and dependent variable as the cognitive measures (i.e., the MMSE scores), and the proposed mediator was DMN gray matter size. Age, gender, and education were controlled as nuisance of no interest. Statistical significance was set at *p* < 0.05.

## Results

### Demographic and Clinical Data

Demographic and clinical data of all the participants are presented in Table 1. We excluded two HHT subjects and one control subject from data analysis due to poor image quality or segment failure; thus, a total of 100 subjects were finally included. The HHT subjects demonstrated an average Hcy of 21.79 ± 10.97 μmol/L compared with the normotensive controls (11.47 ± 7.65 μmol/L, *p* < 0.05). In addition, the HHT subjects demonstrated lower MMSE scores (*p* = 0.006) and higher but non-significant frequency of vascular risks, including diabetes mellitus, hyperlipidemia, and obesity (*p* > 0.05). However, the hypertensive subjects were generally comparable with normotensive controls on age, gender, and education (*p* > 0.05).

**Table 1:** Clinical and demographic information.

**Variable**	**HHT**	**Controls**	**Statistic**, ***t*** **(*****p*****)**
**Demographic data**			
Age, mean (SD), years	53.20 (10.98)	50.84 (9.92)	1.128, 0.26
Sex/gender: males/females	26M/24F	18M/32F	2.60, 0.11
Education, mean (SD), years	11.68 (2.35)	11.46 (2.84)	0.338, 0.51
History of smoking: yes, *n* (%)	24 (48%)	16 (32%)	2.67, 0.10
Obesity: obese, *n* (%)	11 (22%)	5 (10%)	2.68, 0.10
Alcohol	28 (56%)	21 (42)	1.96, 0.16
MMSE	27.22 (0.89)	27.74 (0.96)	−2.805, *p* = 0.006
BP Systolic	159.86 (17.33)	117.92 (7.50)	15.593, *p* < 0.001^***^
BP Diastolic	98.86 (14.83)	77.71 (5.85)	9.312, *p* < 0.001^***^
Hcy (μmol/L)	21.79 (10.97)	11.47 (7.65)	5.462, *p* < 0.001^***^
BMI	24.42 (3.05)	23.48 (2.60)	1.658, 0.102

*** *p < 0.001; SD, standard deviation; MMSE, Mini Mental State Examination; BP, blood pressure; Hcy, homocysteine; BMI, body mass index; HHT, hypertension with high homocysteine*.

### Voxel-Wise Gray Matter Volume

**Figure 1:**
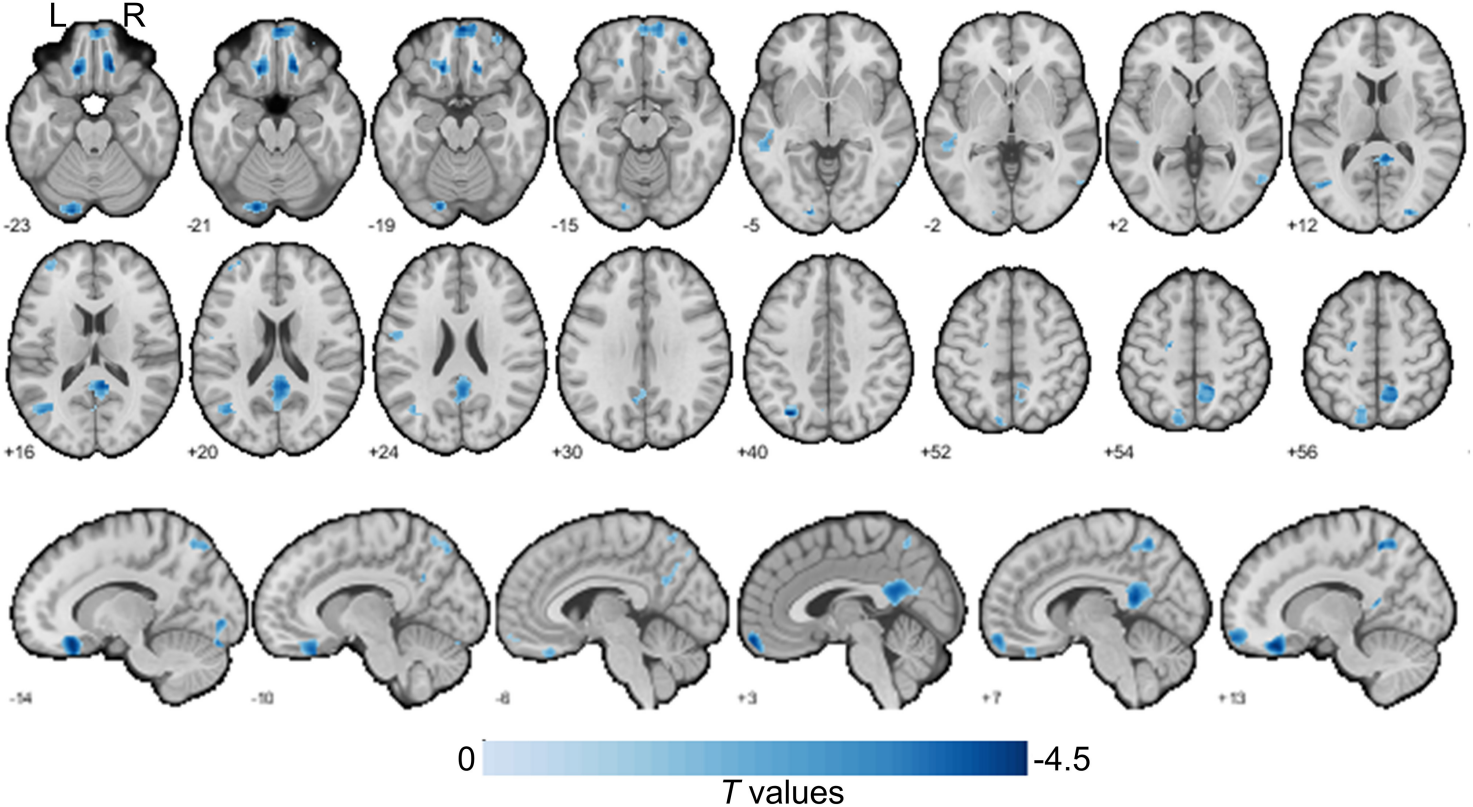
Gray matter size difference between the H-type hypertensive and normotensive subjects. The axial slices are labeled with Z coordinates and sagittal slices with Y coordinates. The results were reported with voxel-wise *p* < 0.001 and family-wise error (FWE) cluster corrected at cluster-level *p* < 0.05. A negative colorbar indicates gray matter losses in the H-type hypertensive subjects. Regions were automatically reported and labeled using BSPMVIEW (http://www.bobspunt.com/software/bspmview) and automated anatomical labeling atlas 3 (https://www.gin.cnrs.fr/en/tools/aal/). L, left; R, right.

To investigate gray matter volume differences, we performed voxel-based morphological analysis. Our findings showed that HHT subjects exhibited smaller gray matter volume in several clusters in the DMN, including the left inferior parietal, right medial prefrontal, precuneus, and bilateral middle temporal gyri and additionally in the left lateral frontal, left precentral, and right superior occipital and left cerebellum crus regions [Table 2, Figure 1; voxel-wise *p* < 0.001 and family-wise error (FWE) cluster corrected at cluster-level *p* < 0.05].

**Table 2:** Gray matter VBM results.

**Brain regions**	**Cluster size**	***t*** **-value**	**MNI coordinates**		
			***x***	***y***	***z***
Parietal_Inf_L	1,334	4.157	−33	−69	41
Rectus_R	1,685	4.068	13	32	−22
OFCmed_L	2,201	4.065	−16	30	−19
Ventral medial prefrontal	2,423	3.994	2	62	−19
Cerebellum_Crus1_L	1,515	3.941	−19	−87	−21
Precuneus_R	4,548	3.913	6	−46	16
Precuneus_R	2,601	3.810	12	−49	56
OFCant_R	676	3.556	31	54	−14
Occipital_Sup_R	478	3.527	26	−90	13
Temporal_Mid_L	1,418	3.463	−45	−67	14
Parietal_Sup_R	343	3.449	28	−60	64
Frontal_Sup_2_L	392	3.367	−20	−13	55
Frontal_Mid_2_L	363	3.260	−41	53	20
Precentral_L	417	3.160	−49	−3	23
Temporal_Mid_L	1,132	3.138	−52	−34	−4
Temporal_Mid_R	405	2.983	55	−64	3

*Regions were automatically labeled using the AAL3 atlas. These results were reported with voxel-wise p < 0.001 and family-wise error (FWE) cluster corrected at cluster-level p < 0.05. Regions were automatically reported and labeled using BSPMVIEW (http://www.bobspunt.com/software/bspmview) and automated anatomical labeling atlas 3 (https://www.gin.cnrs.fr/en/tools/aal/)*.

*MNI, Montreal Neurological Institute; x, y, and z, MNI coordinates in the left–right, anterior–posterior, and inferior–superior dimensions, respectively*.

### Brain Size and White Matter Lesion Burden

The HHT subjects showed larger intracranial volume (*p* = 0.042) and cerebrospinal fluid volumes (*p* = 0.049), and higher WMH burden (*p* = 0.015), but comparable gray matter and white matter sizes (Table 3).

**Table 3:** Gross segment volumes and white matter hyperintensity burden in the two groups included in this study.

**Variable**	**HHT**	**Controls**	**Statistic**, ***t*** **(*****p*****)**
TIV (ml)	1,379.93 (129.08)	1,325.52 (134.79)	2.061, 0.042^*^
GM (ml)	582.94 (57.34)	573.30 (55.58)	−0.854, 0.40
WM (ml)	508.59 (61.56)	487.42 (60.91)	1.728, 0.087
CSF (ml)	286.62 (61.19)	263.61 (53.80)	1.996, 0.049^*^
WMH (ml)	1.78 (1.45)	1.19 (0.87)	−2.484, 0.015^*^

* *p < 0.05; HHT, hypertension with high homocysteine; TIV, total intracranial volume; GM, gray matter; WM, white matter; CSF, cerebrospinal fluid; WMH, white matter hyperintensity*.

### Association Analyses

To determine the relationships between brain (WMH and gray matter volume) and behavioral and physiological parameters, we conducted linear correlation analyses. Results showed that gray matter in the DMN and thalamus was negatively correlated with WMH but positively correlated with MMSE scores. In other words, the DMN gray matter size predicted white matter lesion load and general cognition, although there was no significant relationship between WMH and MMSE.

**Figure 2:**
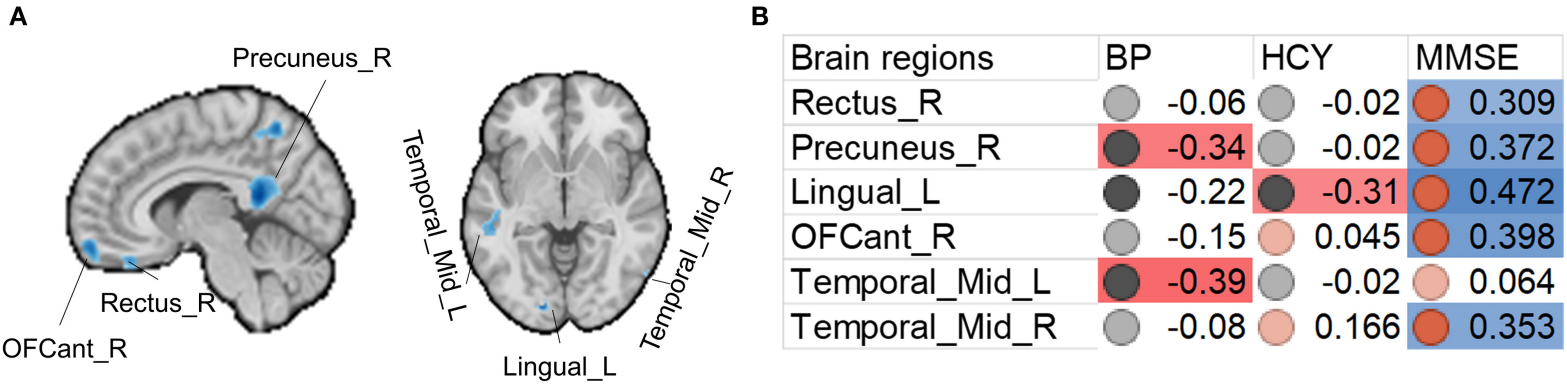
Associations between gray matter size within the significant clusters and clinical (blood pressure), physiological (Hcy level), and behavioral (MMSE scores) parameters in H-type hypertensive subjects. **(A)** Among the significant clusters, brain regions showing at least one significant correlation **(B)** are marked out. **(B)** Heatmap showing Pearson's correlation coefficients between gray matter size within the significant clusters and clinical (blood pressure), physiological (Hcy level), and behavioral (MMSE scores) parameters in H-type hypertensive subjects. Significant correlations are marked with warm (positive) or cold (negative) colors, and non-significant correlations are marked with white color. BP, blood pressure based measure, here hypertension grades 1–3; HCY, homocysteine; MMSE, Mini-Mental State Examination.

**Figure 3:**
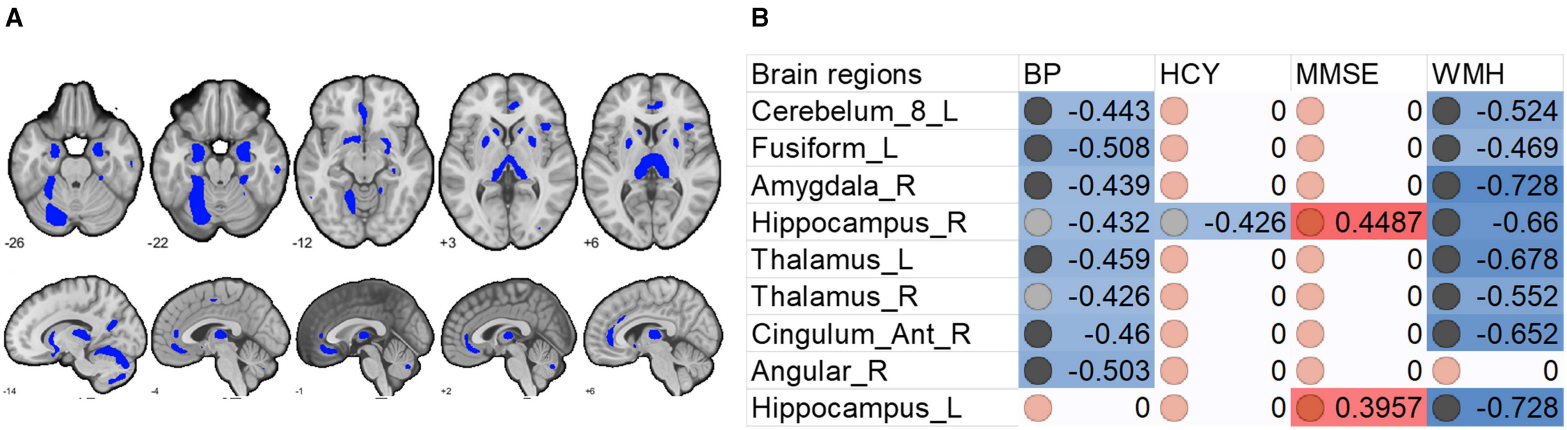
Associations between whole brain gray matter size (excluding the significant clusters) and clinical (blood pressure), physiological (Hcy level), and behavioral (MMSE scores) parameters, and lesion (WMH load) in H-type hypertensive subjects. **(A)** Excluding the significant clusters, brain regions showing at least one significant correlation **(B)** are marked out. **(B)** Heatmap showing Pearson's correlation coefficients between gray matter size within the significant clusters and clinical (blood pressure), physiological (Hcy level), and behavioral (MMSE scores) parameters, and lesion (WMH load) in H-type hypertensive subjects. Significant correlations are marked with warm (positive) or cold (negative) colors, and non-significant correlations are marked with white color. BP, blood pressure based measure, here hypertension grades 1–3; Hcy, homocysteine; MMSE, Mini-Mental State Examination; WMH, white matter hyperintensity.

We also found that increased systolic blood pressure was associated with decreased gray matter size within the thalamus and DMN (Figures 2, 3). We also determined correlations between the whole brain (excluding the significant clusters) voxel-wise gray matter volume and blood pressure (BP), MMSE, Hcy, and WMH loads (Figure 3).

### Mediation Analyses

To investigate whether the gray matter loss significantly affected the association between Hcy and cognitive performance, we used a mediation model to answer this issue. Unfortunately, we did not find any mediation effect of gray matter volume on the reported significant clusters.

## Discussion

In this study, we investigated gray matter atrophy underlying HHT and the associations with cognitive performance and plasma Hcy, while considering multiple vascular variables. We found that HHT patients showed significant DMN atrophy, which can reflect Hcy level, global cognitive function (MMSE), and WMH load. HHT patients demonstrated parallelized gray matter atrophy and global cognitive decline, suggesting that both hypertension and high Hcy aggravate the neuroanatomical and cognitive toxic effects.

Consistent with our hypothesis and previous published reports (16, 35), we found significant gray matter loss in the DMN and sensorimotor regions in the HHT subjects. The DMN has been well-characterized in advanced cognitive functions, including autobiographical memory, social cognition, memory consolidation, and reasoning; for more details, please see Buckner and DiNicola (9) and Raichle (10). Brain regions that constitute the DMN include the inferior parietal lobule that is associated with spatial orientation, the medial prefrontal cortex that is linked to reasoning, precuneus that is linked to social cognition, and middle temporal region that is involved in multimodal sensory processing and social cognition. These components are crucial to advanced cognitive functions (43), have been linked to cognitive impairment in Alzheimer's disease (37, 45), and also generally align to the findings from rodents and other animals (39, 46) in HHT. Anatomically, lateral temporal regions receive anterior circulation whose arteries emerge from the Sylvian fissure, thus a potential focus that is targeted by the hypertension-induced hypoperfusion. Also, these key DMN regions are a marker for cognitive impairment and heavily hit regions of altered hemodynamics. Thus, the DMN atrophy reported here is consistent but beyond the previous published reports on sole or mixed primary hypertension.

Apart from the DMN, we also identified gray matter loss in the left lateral prefrontal, left precentral, right occipital, and cerebellum regions. These regions are mainly involved in sensory and motor processing. For example, the left inferior frontal region is a classical Broca's area, responsible for speech and other higher motor functions, and is also a vulnerable foci for stroke and other cerebrovascular events (47). The precentral region is involved in the somatomotor system and associated with physical fitness and well-being (48, 49). The occipital is also largely a primary sensory cortex and involved in visuospatial processing. Visuospatial deficit is also supposed as a biomarker of Alzheimer's disease (50).

Beyond the gray matter atrophy, we also found significant associations. We first report the associations between significant atrophic clusters with blood pressure, Hcy, and MMSE scores. Gray matter volume in the precuneus and left middle temporal gyrus was negatively correlated with blood pressure; gray matter volume in the left lingual cortex was negatively correlated with plasma Hcy. Nevertheless, we found that the MMSE scores were correlated with almost all the significant clusters, thereby suggesting the role of these regions in global cognition. This finding is in line with many studies investigating the brain anatomical correlates with global cognition (25–30). The precuneus is a core of the DMN, and this result is also in line with other published reports on vascular cognitive impairment (51–53). The middle temporal gyrus is a vulnerable locus attacked by the vascular pathologies. For example, in patients with carotid stenosis, the same region is the most impacted (51–53).

The finding that HHT showed higher WMH load and correlated with lower gray matter is generally consistent with previous reports. For example, previous cohort studies have reported positive associations between elevated Hcy and WMH and silent infarction (54–56) but inconsistent findings on whether these factors act as mediators (54). However, in healthy aging and some major brain diseases, WMH load tends to associate with regional gray matter atrophy (57, 58).

Hcy and cognitive impairment together promote brain atrophy, especially when they cross a threshold level (40). Therefore, we were also interested in investigating whether there is a relationship between high Hcy and cognitive decline mediated by gray matter atrophy in HHT subjects. Unfortunately, due to the lack of a significant correlation between Hcy and global cognition, our mediation model could not confirm this hypothesis. One possibility is that Hcy is a non-specific measure, especially when it is associated with non-specific global cognitive measures such as MMSE. Another possibility, as mentioned at the beginning of this paragraph, is that Hcy needs to reach a certain threshold level to establish a parallel relationship with cognitive impairment, which makes this problem more complicated. In addition, HHT, as a kind of vascular cognitive impairment, may need more sensitive tests focused on specific cognitive domains (59–63). We also found slightly elevated Hcy in the controls, suggesting independent risk factor in this population. Although this does not necessarily provide any indications, it suggests a possibility that there may be regional and dietary predisposition in this population, which may also be the reason for the high incidence of HHT (64). Nevertheless, investigations on this topic are still lacking. However, HHT subjects demonstrated far higher Hcy; this may become highly unfavorable.

We found that the HHT group had larger intracranial and cerebrospinal fluid volumes. One reason is that the HHT group had a higher proportion of males, although there was no statistical difference in the gender ratio; thus, we included gender as a nuisance covariate in all the analyses. At the same time, patients with hypertension have more WMHs, which is consistent with clinical observation and previous published report (24), thereby suggesting that HHT is accompanied by more white matter damage burden than the normotensive controls.

Although we have reported some novel findings, several limitations of our study cannot be neglected. First, we could not build the casual relationship between Hcy and hypertension, which might affect the statistical model; nonetheless the current findings suggest dual adverse effects from HHT. Further study may validate the casual relationship by considering more variables, including WMH load, blood pressure classification, vitamins, and other risk factors. Second, factors affecting gray matter atrophy also include hemodynamic changes (65) and lacunar infarction (66–68). Although these two aspects are still poorly characterized, they may contribute to brain atrophy to varying degrees. Future research should include these factors as explanatory variables and promote a more comprehensive model. Third, we only measured global cognition for each subject; this could hamper the ability to classify between-group differences. Increasing evidence suggests that vascular cognitive impairment is more likely to affect processing speed and semantic/recall memory, which is different from the pattern of global cognitive impairment caused by Alzheimer's disease (60, 69–71). Therefore, we need to further include domain-specific tests, such as the Digit Symbol (72) and the Rey Auditory Verbal Learning Tests (73), to accurately characterize the affected cognitive domains that are most sensitive to HHT and its underlying neuroanatomical and physiological basis.

## Conclusion

In conclusion, this study reports that HHT is associated with default network atrophy and correlated with WMH, Hcy level, and MMSE score. These results provide, to our knowledge, the first insights into how HHT affects brain gray matter morphology and how these three risk factors interact with one another.

## Data Availability Statement

The original contributions presented in the study are included in the article/supplementary material, further inquiries can be directed to the corresponding author.

## Ethics Statement

The studies involving human participants were reviewed and approved by the Ethical Committee for Human Research at the People's Hospital of Tongchuan City. The patients/participants provided their written informed consent to participate in this study.

## Author Contributions

YK collected data and wrote the draft. XL wrote the draft. LC, YL, and LJ collected data and analyzed data. LG analyzed data and proofread the manuscript. LR conceived and revised the manuscript. All authors contributed to the article and approved the submitted version.

## Funding

This work was supported by the Natural Science Foundation of China (Grant No. 82001799).

## Conflict of Interest

The authors declare that the research was conducted in the absence of any commercial or financial relationships that could be construed as a potential conflict of interest.

## Publisher's Note

All claims expressed in this article are solely those of the authors and do not necessarily represent those of their affiliated organizations, or those of the publisher, the editors and the reviewers. Any product that may be evaluated in this article, or claim that may be made by its manufacturer, is not guaranteed or endorsed by the publisher.
